# 

**Published:** 1880-12

**Authors:** 


### CONTENTS.

### COMMUNICATIONS.

Some Points in the Natural History of Caries of the Teeth, and the Value of Fillings for its Arrest .......... 515
Saliva—Its Characteristics in Health and Disease .......... 526
A Short Paper on Nervous Reflex Action .......... 532
How to Distinguish Oleomargarine .......... 541
Our Duty .......... 542
The Apostles Creed .......... 544

### SELECTIONS.

The Voice .......... 545
New Substitute for Rubber .......... 546
Swarming Extraordinary .......... 547
Another Mastodon .......... 547
Easy Test for Arsenic in Fabrics .......... 548
An American Elephant .......... 548
On Vaccination .......... 548
Children's Hats .......... 549
On the Absolute Invisibility of Atoms and Molecules .......... 550
Fate of the Discoverers of Anæsthesia .......... 552
The Rubber Patents .......... 552
Correspondence .......... 553
Clinic Reports .......... 554

### EDITORIAL.

The Scientific American .......... 556
A New "Special" Pattern Tooth Brush .......... 557
Physicians' Visiting List .......... 557
Obituary .......... 558

